# In *Salmonella enterica*, OatA (Formerly YjgM) Uses *O-*Acetyl-Serine and Acetyl-CoA to Synthesize *N,O-*Diacetylserine, Which Upregulates Cysteine Biosynthesis

**DOI:** 10.3389/fmicb.2018.02838

**Published:** 2018-11-27

**Authors:** Chelsey M. VanDrisse, Jorge C. Escalante-Semerena

**Affiliations:** Department of Microbiology, University of Georgia, Athens, GA, United States

**Keywords:** *N*, O-diacetylserine, *O*-acetyl-serine, cysteine biosynthesis, *N-*acetyltransferases, YjgM, small molecule acetylation, gene expression control

## Abstract

L-Cysteine biosynthesis has been extensively analyzed in *Salmonella enterica*. The cysteine regulon contains the genes whose protein products are necessary to convert sulfate to sulfide, which is eventually reacted with *O*-acetyl-serine (OAS) to generate cysteine. The LysR type regulator, CysB, is required for activation of the cysteine regulon, and its interaction with various *cys* genes has been thoroughly characterized. Results from previous studies by others, suggested that OAS undergoes a spontaneous *O*- to *N-* migration to produce *N*-acetyl-serine (NAS), and that NAS is the true signal sensed by CysB. It was unclear, however, whether such migration occurred spontaneously *in vivo* or if NAS was generated enzymatically. Work reported herein characterizes a *S. enterica N*-acetyltransferase, OatA (formerly YjgM), which acetylates the *N*_α_-amino group of OAS, producing *N,O-*diacetyl-serine (DAS) at the expense of acetyl-CoA. We isolated OatA to homogeneity and performed its initial biochemical characterization. The product of the OatA reaction was isolated by HPLC and confirmed by mass spectrometry to be DAS; OatA did not acetylate NAS, consistent with the conclusion that OatA is an *N-*acetyltransferase, not an *O-*acetyltransferase. Binding of OAS to OatA appears to be positively cooperative with the apparent *K_0.5_* for OAS determined to be 0.74 mM, the *k_cat_* was 1.05 s^-1^, and the catalytic efficiency of the enzyme (*k*_cat_/*K_0.5_*) was 1.4 × 10^3^ M^-1^ s^-1^. Size exclusion chromatography indicated that OatA was a monomer in solution. In *S. enterica*, overexpression of *oatA* led to shorter lag times on sulfate-limiting medium and that these delayed lag times were due to increased expression of the cysteine regulon, as indicated by RT-qPCR results. OatA is the first Gcn5-related *N-*acetyltransferase (aka GNAT) involved in the regulation of amino acid biosynthetic genes in *Salmonella*. On the basis of results of transcriptomics studies performed by other investigators, we hypothesize that DAS may play a role in biofilm formation in *S. enterica* and other bacteria.

## Introduction

**FIGURE 1:**
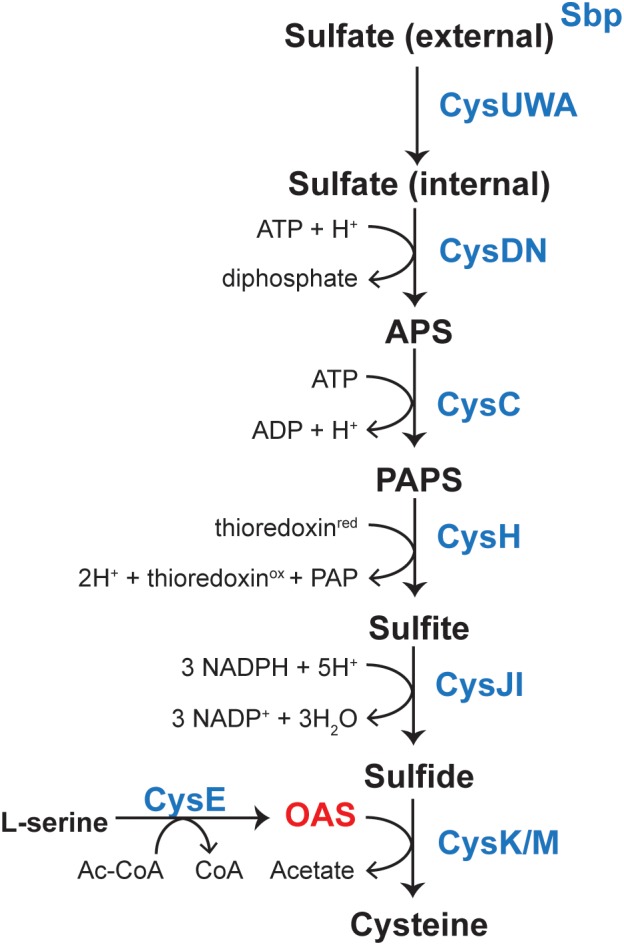
Pathway for biosynthesis of L-Cys in *Salmonella enterica.* Sulfate is sequestered by the sulfate-binding protein (sbp) and uptake is mediated by the CysUWA sulfate/thiosulfate transporter. The ATP sulfurylase [CysD (catalytic subunit)/CysN (GTP-binding subunit), EC 2.7.7.4] activates sulfate to adenosine 5′-phosphate (APS), which is a substrate for APS kinase (CysC, EC 2.7.1.25). 3-phosphoadenosine 5′-phosphosulfate (PAPS) is reduced to sulfite by the PAPS reductase (CysH, EC 1.8.4.8) and sulfite is reduced to sulfide by a NADPH-sulfite reductase (CysJI, EC 1.8.1.2). A serine acetyltransferase (CysE, EC 2.3.1.30) produces OAS from L-Ser and AcCoA. OAS and sulfide are then substrates for cysteine synthase (CysK, EC 2.5.1.47), which produces L-Cys. Transcription of *cysJIH* and *cysK* is upregulated upon binding of CysB and acetyl-serine.

L-Cysteine (L-Cys) is one of the 22 proteinogenic amino acids in nature, and one of only two amino acids whose side chains contain a sulfur atom. The sulfhydryl group of L-Cys is critical to maintaining redox homeostasis in cells from all domains of life (Hernandez et al., 2015; Hasan et al., 2016; Zhang et al., 2016; Cao et al., 2017; Liu et al., 2018; Tang et al., 2018). The biosynthesis of this important amino acid has been extensively studied (Kredich, 2008). *S. enterica* synthesizes L-Cys *de novo* using a pathway that reduces sulfate (oxidation state 6^+^) to sulfide (oxidation state 2^-^) and then condenses *O*-acetyl-serine (OAS) with sulfide to yield L-Cys and acetate (Figure 1) (Kredich, 2008). Expression of several genes encoding enzymes of the pathway are regulated by CysB, a LysR type regulator (Jagura-Burdzy and Kredich, 1983; Ostrowski et al., 1987). CysB has a complex mechanism of transcriptional regulation. Previous literature has characterized binding of CysB to the promoter regions of *cysK* [encodes *O*-acetylserine (thiol)-lyase, EC 2.5.1.47], *cysJIH* (encode sulfite reductase, EC 1.8.7.1), *cysP* (encodes thiosulfate-binding protein), and to *cysB* (encodes a LysR regulator) (Monroe et al., 1990; Hryniewicz and Kredich, 1991, 1994; Ostrowski and Kredich, 1991).

**FIGURE 2:**
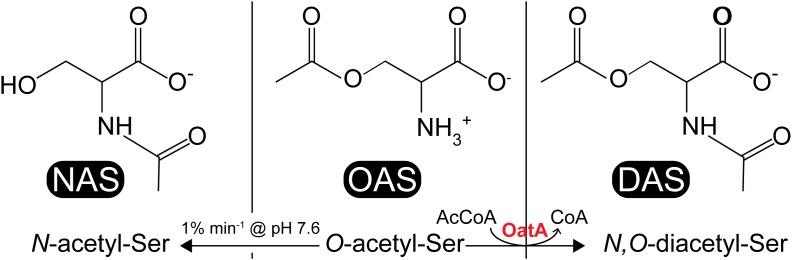
Acetyl-serine analogs: NAS, OAS, and DAS. NAS (left) is thought to be a ligand for CysB, which is produced from non-enzymatic *O*-to-*N* intramolecular migration of OAS (center) at ≥pH 7.6 at a rate of 1% min^-1^. DAS (right) is produced from acetylation of OAS by the *S. enterica* OatA *N*-acetyltransferase.

In the literature, CysB binding to its target promoters was reported to be allosterically modulated by *N*-acetyl-serine (NAS) and OAS. For years, OAS, the product of CysE (serine transacetylase, EC 2.3.1.30) and a substrate for CysK (Figure 1), was suspected to be the inducer of the cysteine regulon (Jones-Mortimer et al., 1968; Kredich, 1971). However, subsequent studies suggested that the more likely inducer was NAS (Ostrowski and Kredich, 1989). *In vitro*, NAS is produced from intramolecular *O* to *N* migration of the acetyl moiety, which was suggested to occur at a very fast rate (1% per minute at pH 7.6 (Flavin and Slaughter, 1967) (Figure 2). It was not known, however, whether this conversion was enzymatically catalyzed *in vivo*. Regardless, studies characterizing CysB ligand and promoter interactions utilized OAS as the source of NAS and referred to the ligand as acetyl-Ser (Monroe et al., 1990; Hryniewicz and Kredich, 1994; Kredich, 2008).

*In vitro* electrophoretic mobility shift assays (EMSAs), DNA footprinting, and *in vitro* transcription assays revealed *cis* activation regions in *cysJIH, cysK*, and *cysP*. CysB binding to these regions was apparently altered upon interaction of CysB with acetyl-Ser (Kredich, 2008). In brief, CysB is a complex regulator that binds to accessory and activation sites of *cysK, cysJIH, cysP*, and *cysB* promoters and its binding to these sites is altered by acetyl-Ser. Molecular details on how this change in CysB promoter binding alters transcription remains unclear, although models that predict changes in DNA bending are thought to be at play (Hryniewicz and Kredich, 1991, 1994, 1995).

The *S. enterica* genome encodes up to 26 Gcn5-related putative *N*-acetyltransferases (GNATs, PF00583), but not all of them have been characterized (Hentchel and Escalante-Semerena, 2015a). Some *N*-acetyltransferases target *N*_∊_-amino groups of lysyl residues (Burckhardt and Escalante-Semerena, 2017; VanDrisse et al., 2017), others modify *N*_α_-amino groups of proteins (Rycroft et al., 2018), and yet a third class targets the *N*_α_-amino groups of small molecules (Hentchel and Escalante-Semerena, 2015b). In general, acetylation changes the chemical properties of the substrate by modulating the activity of the protein target or altering the way a small molecule interacts with its target.

Our interest in OAS as a target for acetylation was prompted by results obtained using a broad small molecule acetylation substrate screen (Kuhn et al., 2013). The screen was performed using purified uncharacterized *N-*acetyltransferases from *S. enterica* (STM1857, ElaA, YafP, YhbS, YiiD, YjaB, YjgM, and YpfL). From these experiments we showed that YjgM (a putative acetyltransferase) acetylated OAS *in vitro*, a product we suspected to be *N,O-*diacetyl-serine (DAS, Figure 2). Herein, we report the initial biochemical characterization of YjgM and show that this protein acetylates OAS converting it to DAS at the expense of acetyl-CoA (AcCoA). We also report RT-qPCR data that support the conclusion that OatA activity enhances growth under conditions that require cysteine biosynthesis, and that overexpression of *oatA* leads to an increase in transcription of the cysteine regulon. Relevant to our work is the report by others that *oatA* expression increases during planktonic growth when compared to cells in a biofilm (MacKenzie et al., 2015). At present, it is unclear whether the positive effects of DAS on cysteine biosynthesis are direct or indirect. Based on the biochemical data reported here, we suggest changing the name of the protein from YjgM to OAS acetyltransferase A or OatA, and that the *yjgM* gene now be referred to as *oatA.*

## Materials and Methods

### Bacterial Strains, Culture Media, and Chemicals

All bacterial strains are listed in Table 1. All strains for growth analysis were derivatives of *Salmonella enterica* subsp. *enterica* serovar Typhimurium LT2 (hereafter *S. enterica*). *S. enterica* strains were grown at 37°C on lysogeny broth (LB, Difco) or no-carbon essential (NCE) minimal medium (Berkowitz et al., 1968) supplemented with glycerol (22 mM), MgSO_4_ (concentrations varied and noted in the figure legends), and Wolfe’s trace minerals (1x) (Atlas, 1995). *Escherichia coli* C41 (λDE3) (Miroux and Walker, 1996) was used for protein overexpression and *E. coli* DH5α (New England Biolabs) was used for plasmid construction. All *E. coli* strains were grown at 37°C in LB. Antibiotics for all media were used at the following concentrations: ampicillin, 100 mg mL^-1^; chloramphenicol, 20 mg mL^-1^. All chemicals were purchased from Sigma-Aldrich unless otherwise noted; isopropyl β-D-1-thiogalactopyranpside (IPTG, Gold BioTechnology), glycerol (Fisher Scientific), [1-^14^C] acetyl-CoA (Moravek, Inc.), HEPES (Gold BioTechnology). OAS and L-CysHCl solutions were made fresh in HEPES (pH 7 at 25°C) immediately before each *in vitro* or *in vivo* experiment.

**Table 1:** Bacterial strains used in this study.

Strain	Relative genotype	Source^1^
***E. coli strains***		
*E. coli* DH5α	Φ80d*lacZ*DM15 *recA*1 *endA*1 *gyrA*96 *thi*-1 *hsdR*17 (r_k_-, m_k_^+^) *supE*44 *relA*1 *deoR* Δ(*lacZYA-argF)* U169 *phoA*	NEB
*E. coli* C41 (λDE3)	*pka12*::*kan^+^ ompT hsdS* (r_B_m_B_) *gal* (λDE3*)*	Laboratory collection
***S. enterica strains***		
JE10079	*ara-9*	Laboratory collection
Derivatives of JE10079		
JE23873	*oatA^+^*/pCV3	
JE23874	*oatA*::*kan*^+^/pCV3	
JE23875	*oatA*::*kan*^+^/p*oatA^+^*	
JE19460	Δ*cysE*/pCV3	
JE24366	*cysB*::*kan*^+^/pCV3	


*^*1*^Unless otherwise noted, all strains were constructed in this study.*

### Plasmid Construction

All plasmids used in this work are listed in Table 2. Primers were synthesized by Integrated DNA Technologies, Inc. (IDT [Coralville, IA, United States]) and are listed in Table 3. Genes were amplified from *S. enterica* genomic DNA using Pfu Ultra II fusion DNA polymerase (Stratagene) per manufactures instructions. A high efficiency cloning method described elsewhere (Galloway et al., 2013; VanDrisse and Escalante-Semerena, 2016) was used to clone *oatA* and *cysB* genes into pCV3 (complementation vector, L-(+)-arabinose inducible) and pTEV16 or pTEV18 (overexpression vectors, IPTG inducible). Restriction enzyme BspQI was purchased from New England Biolabs. The resulting complementation vector was referred to as pOatA6. The resulting overexpression vectors were referred to as pOatA4 and pCysB2 (full length CysB).

**Table 2:** Plasmids used in this study.

Plasmid	Genotype	Source^1^
pTEV16	*lacI^+^ bla^+^*	VanDrisse and Escalante-Semerena, 2016
pTEV20	*lacI^+^ bla^+^*	VanDrisse and Escalante-Semerena, 2016
pCV3	*araC^+^ cat^+^*	VanDrisse and Escalante-Semerena, 2016
**Overexpression plasmids**		
pCysB2	*S. enterica cysB*^+^ cloned into pTEV20, *bla^+^*	
pOatA4	*S. enterica oatA*^+^ cloned into pTEV16, *bla^+^*	
**Complementation plasmids**		
pOatA6	*S. enterica oatA*^+^ cloned into pCV3, *cat^+^*	


*^*1*^Unless otherwise noted, all plasmids were constructed in this study.*

**Table 3:** Primers used in this study.

Primer name	Primer sequence 5′ → 3′
**Overexpression primers**	
5′ *oatA* pTEV16	NN**GCTCTTC**NAGCATGAATAATGTCGCCTCGCCAACGC
3′ *oatA* pTEV16	NN**GCTCTTC**NTTATCAGAGATCCTTTAACATCCTCACTTCG
5′ *cysB* pTEV20	NN**GCTCTTC**NTTCATGλTTGCAGCAGCTTCGTTAC
3′ *cysB* pTEV20	NN**GCTCTTC**NTTATTACTTTTCAGGCAGCTTTATATCCTG
**Complementation primers**	
5′ *oatA* pCV3	NN**GCTCTTC**NTTCATGAATAATG TCGCCTCGCCAACGC
3′ *oatA* pCV3	NN**GCTCTTC**NTTATCAGAGATCCTTTAACATCCTCACTTCG
***S. enterica* wanner deletion primers**	
5′ *cysB* wanner *kan*^+^	ACGATGGCCTGATGGCGCTAATCTGGATGATGTATT GTGTAGGCTGGAGCTGCTTC
3′ *cysB* wanner *kan*^+^	GTCTGCCATGCCACTACGACACλCCGACGGTGAT ACATATGAATATCCTCCTTAG
5′ *cysE* wanner *kan*^+^	AACGGGTTGGTCGTTTTCTGCCCGTCTGGAGTAAGC CATGGTGTAGGCTGGAGCTGCTTC
3′ *cysE* wanner *kan*^+^	CTCCATCGGAACAGCGTTTTTTAGTTGTACCGCGCA ATTCACATATGAATATCCTCCTTAG
5′ *oatA* wanner *kan*^+^	GTCAGCTTCCGGCGTGGCCGCGGATAACAAGAGA GAGTGTAGGCTGGAGCTGCTTC
3′ *oatA* wanner *kan*^+^	AGATGCCTTCATCGAGTAGTTGGATATGTCCAGCT ACATATGAATATCCTCCTTAG
**Electrophoretic mobility shift assay primers**	
5′ *cysJIH*	TCCAACCCTTCTTTAATTGTTATTCCTC
3′ *cysJIH*	/56-FAM/CGTCATGCGTCGTTATGTTCCAG
**RT-qPCR primers**	
5′ *cysB*	AAGCGGTATCGλGGGAAC
3′ *cysB*	TAGCATGGCAACATCACCAG
5′ *cysD*	GCTTCCGTACACTTGGCTGT
3′ *cysD*	CTGATCGCGGTCAATCATC
5′ *cysI*	CCGCCCGTATGAGTTTACC
3′ *cysI*	AGCGTCAGGTGCCATTTATC
5′ *cysK*	TGCGGTAGAACCCACTGACT
3′ *cysK*	GGCCTGGTTTGATCTCTTCA
5′ *cysM*	TACGCCCACTACACGACCAC
3′ *cysM*	GAATGCCCGGAATACTGCT


*BspQI sites are indicated in boldface.*

### Strain Construction and Growth Analysis

In frame deletions of *S. enterica* genes were constructed using the phage lambda Red recombinase system as described (Datsenko and Wanner, 2000; VanDrisse et al., 2017). Plasmids were transformed into strains for complementation studies as described (VanDrisse et al., 2017). For growth analyses, starter cultures were grown overnight at 37°C shaking in LB containing appropriate antibiotic. Cultures (1% [v/v]) were used to inoculate 198 μL of fresh minimal medium placed in each well of a 96-well microtiter plate. When necessary before inoculation, cells were washed (x 2) with NCE (1x) by centrifuging 1 mL of culture at 6,000 × *g* for 3 min. Figure legends indicate whether inoculum was greater than 1% (v/v). L-(+)-Arabinose was used as inducer when indicated. Under sulfur-limiting conditions, MgCl_2_ (1 mM) was used instead of MgSO_4_. The following chemicals had varying concentrations depending on experiment and are indicated in the figures or figure legends: OAS, L-Cys, and MgSO_4_. Microtiter plates were incubated at 37°C inside the temperature-controlled chamber of a PowerWave microtiter plate reader (BioTek Instruments), and plates were continuously shaken using the slow setting of the instrument. Time points were taken for 24 h every 15 min with data shown for every 2 h. Cell density was monitored at 630 nm and data were analyzed using Prism v6 (GraphPad). Growth studies were performed in triplicate in three independent experiments, with a representative growth curve shown with standard deviation of technical triplicates.

### Protein Overproduction and Purification

Plasmids coding for proteins of interest were transformed into *E. coli* C41 (λDE3). Overnight cultures (50 mL) of transformants were sub-cultured (2% [v/v]) into 1 L of LB containing ampicillin. Cultures were grown at 25°C with shaking to an optical density of 0.6 (OD_600nm_) after which plasmid expression was induced by addition of IPTG (0.5 mM) and shaken overnight. Cells were harvested by centrifugation at 6,000 × *g* for 10 min in a Beckman Coulter Avanti J-20 XOI refrigerated centrifuge with a JLA-8.1000 rotor at 4°C. Cell pellets were stored at -80°C until used. For purification, cell pellets were thawed and re-suspended in 30 mL of buffer A (4-(2-hydroxyethyl)-1-piperazineethanesulfonic acid; HEPES [50 mM, pH 7.0 at 4°C], NaCl [500 mM], and imidazole [20 mM]) containing lysozyme (1 mg/mL) DNase (1 μg/mL), and protease inhibitor phenylmethanesulfonyl fluoride (PMSF, 0.5 mM). Cells were lysed by two rounds of sonication on ice [1 min total (2 s on 2 s off, 60% duty)] using a QSonica sonicator. Clarified lysates were obtained after centrifugation for 30 min at 4°C at 40,000 × *g* in a Beckman Coulter Avanti J-25I centrifuge with JA-25.50 rotor followed by filtration of supernatant through a 0.45 μm filter (Millipore). Samples were applied at 4°C to a 1-mL HisPur nickel-nitrilotriacetic acid (Ni-NTA) resin (Thermo Scientific) that was pre-equilibrated with 10 column volumes (CV) of water and 10 CV of buffer A. After lysate was applied to the column, it was washed with 10 CV of buffer A, 5 CV of buffer B (HEPES [50 mM, pH 7.0 at 4°C], NaCl [500 mM], imidazole [60 mM]). Proteins were eluted with 5 CV of buffer C (HEPES [50 mM, pH 7.0 at 4°C], NaCl [500 mM], imidazole [500 mM]). Fractions were run on an SDS-PAGE gel and fractions containing the desired protein were combined and dialyzed for 3 h at 4°C in HEPES (50 mM, pH 7.0) with decreasing concentrations of NaCl (400 mM, 200 mM, and 150 mM) with a final buffer composition of HEPES (50 mM, pH 7.0 at 4°C), NaCl (150 mM), and glycerol (20% v/v). Final protein concentration was determined using the extinction coefficient and molecular weight (ExPASy, ProtParam) of each protein with a NanoDrop. Proteins were drop-frozen in liquid nitrogen and stored at -80°C.

### *In vitro* Acetylation Activity Assays

To determine specific activity and kinetic parameters of OatA for L-Ser, NAS, or OAS, a continuous spectrophotometric assay that employed 5,5′-dithiobis-(2-nitrobenzoic acid) (DTNB, Ellman’s reagent) was utilized as described (Hentchel and Escalante-Semerena, 2015b). Briefly, assays were performed at 25°C in 100 μL reaction volumes in 96-well plates and reactions were monitored at 412 nm. Reaction mixtures contained HEPES buffer (50 mM, pH 7.2), DTNB (0.3 mM), AcCoA (100 μM), protein (1 μg) and substrate (200 μM). To obtain data for enzyme efficiency (*k*_cat_/*K_m_*), AcCoA was added at saturating levels (1 mM) with sweeps of OAS (0–5 mM). Data were acquired using the Soft Max Pro 6.2 Spectramax software every 10 s over 5 min. Path lengths were calculated using Soft Max Pro 6.2 endpoint readings and absorbance data were corrected for path lengths of 1 cm.

Specific activities were calculated from the slope of the linear range of (ΔA_415_ s^-1^) using Beer’s Law (*A* = e*lc*) with a path length of 1 cm and the molar extinction coefficient of the TNB^2-^ thiolate anion (14,150 M^-1^ cm^-1^) (Riddles et al., 1983). The equation was solved for *c*, giving specific activity in mmol min^-1^mg^-1^ of OatA.

To calculate kinetic parameters, graphs of initial velocity (mM s^-1^) *versus* substrate concentration (mM) were plotted using Prism v6 (GraphPad). Data were fitted to sigmoidal equation to determine *K_0.5_* and *V*_max_. The turnover number (*k*_cat_) was determined using the following equation: V*_max_ = k*_cat_[*E*], where [*E*] was the concentration of protein added. All spectrophotometric assays mentioned above were completed thrice, each in technical triplicate with a representative data set shown. Error bars represent standard deviation as calculated by Prism v6 (GraphPad).

### Size Exclusion Chromatography

A Superose 12 10/300 GL gel filtration column (GE Healthcare Life Sciences) was equilibrated as per manufacturer’s protocol using water and buffer (50 mM HEPES, pH 7.0 at 4°C, 150 mM NaCl). Samples were applied to column using a 100-mL superloop. Gel filtration standards (Bio-Rad) were applied to column first and a standard curve was calculated using the MW_log_ of each standard against retention time of each protein. Purified OatA was eluted from the column in the same manner and retention times were recorded and molecular weights were determined using equation calculated from the standard curve.

### HPLC Purification of *N,O*-Diacetyl-L-Serine (DAS)

Reactions containing OatA (80 μM), AcCoA (1.2 mM), OAS (1 mM) and sodium phosphate buffer (pH 7.0 at 25°C) were incubated for 1 h at 37°C. OatA was removed by passing reactions over an Amicon Ultra 0.5 mL 10 kDa molecular cut off centrifugal filter (Millipore). The product of OatA was resolved by HPLC using a Shimadzu Prominence UFLC with an Aminex HPX-87H column (Bio-Rad). The column was equilibrated at a flow rate of 0.5 ml min^-1^ with five column volumes of buffer [5 mM H_2_SO_4_ in water]. Samples (100 μl) were injected and products were resolved isocratically. Compounds were detected and compared to standards at 215 nm using a computer-controlled Shimadzu Nexera X2 SPD-30A diode array detector. Data were analyzed using Prism v6 (GraphPad). Fractions were collected in 0.5 mL increments and fractions containing peaks corresponding to DAS were analyzed by MS (Protein and Mass Spectrometry Facility, UGA, Athens, GA, United States). Electrospray ionization (ESI)-MS was performed in methanol and resolved on an Esquire 3000 Plus (Bruker) Ion Trap Mass Spectrometer at 0.3 ml h^-1^.

### Growth of Cultures for RNA Isolation

For cultures grown under unlimited sulfur conditions, (MgSO_4_ 1 mM), five individual colonies were inoculated into five independent tubes containing LB + Cm (2 mL) and grown overnight at 37°C. Stationary phase ODs were calculated by diluting (1:10) and measuring absorbance (OD_600_). Cells were then inoculated into minimal medium (10 mL, conditions described above) with a starting OD of 0.1. Cultures were grown for 6 h, spun down in conical tubes (15 mL), flash frozen and stored at -80°C until use.

For cultures grown under sulfur-limiting conditions, cells were grown as just described with the following exceptions. Before calculating stationary phase OD, cells were washed (x2) with NCE (1x). Cells were inoculated into minimal medium (two 10 mL cultures) with MgCl_2_ (1 mM) in lieu of MgSO_4_.

### RNA Isolation

Cell pellets were thawed and RNA was isolated using a protocol described elsewhere (Stead et al., 2012). Briefly, cells were re-suspended (150 μL) in boil solution [ethylenediaminetetraacetic acid, EDTA, (18 mM), SDS (0.025% v/v), 2-mercaptoethanol (1% v/v), formamide (95% v/v), RNase-free water (up to final volume)] and heated (95°C) for 7 min. Cell suspensions were spun down (16,000 × *g*) for 5 min at room temperature and supernatant (100 μL) was transferred to a new tube. Solution was diluted with RNase-free water (400 μL) and sodium acetate (50 μL, final concentration 0.3 M). Ice-cold ethanol (95% v/v, 1650 μL) was added and solutions were incubated at -80°C for 1 h. Solutions were centrifuged at 16,000 × *g* for 30 min at 4°C and supernatant was decanted. RNA/DNA pellets were washed with 300 μL cold ethanol (70%, v/v) and centrifuged at 8,000 × *g* for 5 min. Supernatant was decanted, tubes were centrifuged briefly (3,000 × *g* for 30 s) and liquid was aspirated via pipetting. Pellets were air-dried upside down on Kim-Wipes for 30 min and re-suspended in 100 μL RNase-free water. Tubes were centrifuged at 16,000 × *g* for 1 min (to rid of water-insoluble material) and 90 μL was transferred to a new tube for subsequent DNase digestion. DNA was digested using TURBO DNase (Thermo Fischer Scientific) following the protocol for rigorous DNase treatment. After DNase treatment and DNase inactivation, mixtures were centrifuged at 10,000 × g for 1 min and 100 μL of supernatant was transferred to a fresh tube. RNA was sodium acetate/ethanol precipitated as described above. RNA was re-suspended in 100 μL, aliquoted into four tubes and frozen at -80°C for later use. RNA quality and concentration were assessed using the Agilent 2100 Bioanalyzer with the RNA 6000 nano kit (Georgia Genomics Facility, University of Georgia). Samples with RIN values over 6 were considered adequate for subsequent RT-qPCR experiments.

### cDNA Synthesis and Quantitative Reverse Transcription PCR

RNA samples were diluted in RNase free water (final concentration 40 ng/μL) and used as template (400 ng RNA final) to synthesize cDNA using the iScript cDNA synthesis kit (Bio-Rad Laboratories) per manufactures protocol. Synthesized cDNA was diluted to 7.5 ng/μL to be used as a template for PCR. For real-time PCR, 20 μL reaction mixtures were prepared in 96-well plates with 10 μL of FastSYBR green master mix (Applied Biosystems), gene-specific primers (500 nM), and cDNA (15 ng or 2 μL of above diluted cDNA). The RT-qPCR was performed using a 7500 Fast real-time PCR system (Applied Biosystems). The *S. enterica gyrB* gene was used as an internal control and gene-specific primers were designed using Primer 3 software. Each biological replicate (three for each strain) was analyzed in technical triplicate. Cycle threshold (*C_T_*) data were normalized to *gyrB* and these values were transformed using 2(e-D*C_T_*)/10^-6^ (Livak and Schmittgen, 2001) and reported as arbitrary gene expression units (EU). Mean EU values of each technical triplicate were used to calculate the standard error of the mean (SEM) of three biological replicates using the Prism v6 software. Differences between *oatA*^+^ and *oatA*::*kan*^+^/pOatA^WT^ or *oatA*::*kan*^+^/pCV3 and *oatA*:*kan*^+^/pOatA^WT^ were compared using Welch’s *t*-test with GraphPad Prism v6 software.

### Isothermal Titration Calorimetry (ITC)

Protein (OatA) was purified as described above and concentrated to at least 100 μM before dialyzing three times in filtered HEPES (50 mM, pH 7.0 at 25°C). The final dialysis buffer was saved and used to clean and equilibrate the cell and syringe system, for re-suspension of ligands, and dilution of protein. OatA was diluted to 50 μM and added to the ITC cell following instruments instructions. AcCoA (1 mM) or OAS (0, 0.5, 1, 2, and 20 mM) was re-suspended in dialysis buffer and used as titrant (50 μL) in the stirring syringe. After cell and syringe were loaded, stirring (350 rpm) was initiated until the baseline was re-equilibrated. Titration was initiated using a program for 20 injections (2.48 μL each, 300 s apart) with continuous stirring (350 rpm). ITC data were analyzed using NanoAnalyze software (TA Instruments). Each condition was repeated in triplicate with a representative data set shown in figures.

## Results

### The OatA Acetyltransferase Acetylates *O*-Acetyl-Serine

**FIGURE 3:**
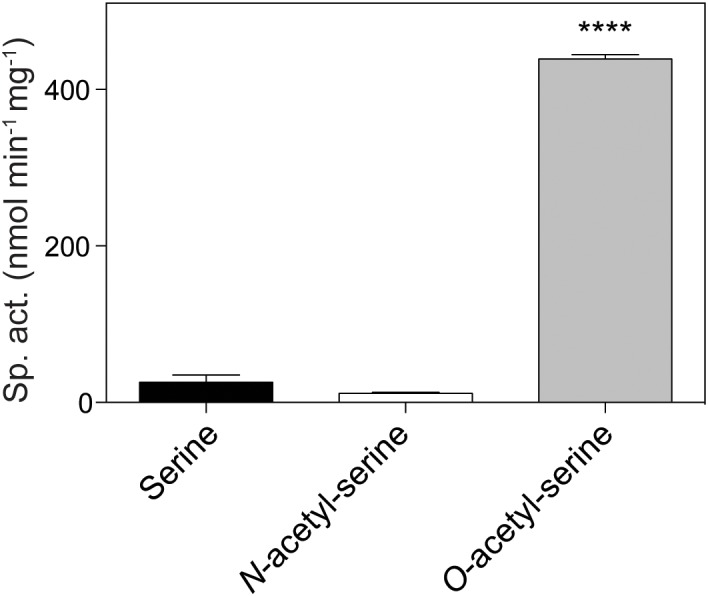
*In vitro* acetylation of *O*-acetyl-serine by OatA. Using a continuous spectrophotometric assay as described under “Materials and Methods” section, OatA (0.5 μM), AcCoA (500 μM), and either L-Ser, *N-*acetyl-serine (NAS), or *O-*acetyl-serine (OAS) (200 μM) were incubated and specific activity for each substrate was calculated. Error bars represent standard deviation and ^∗∗∗∗^ represents a *p*-value < 0.0001.

**FIGURE 4:**
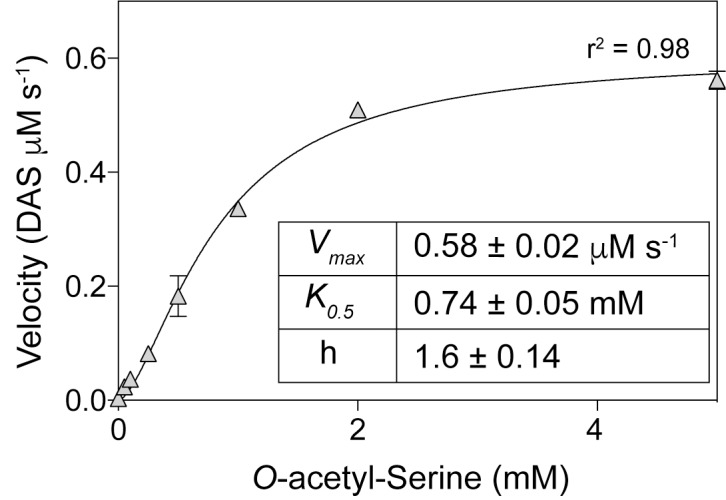
Kinetics of OatA-mediated DAS formation. Using a continuous spectrophotometric assay as described under “Materials and Methods” section, OatA (0.5 μM), AcCoA (1 mM), and *O-*acetyl-serine (OAS) (concentrations indicated on *x*-axis) were incubated and monitored to calculate *V*_max_ at each OAS concentration. *V*_max_ values were plotted against OAS concentrations and an apparent *K_0.5_* was calculated by fitting data to the Hill equation as indicated by black line. Each data point represents the mean of three replicates and error bars represent standard deviation. Experiment was repeated in triplicate with a representative curve shown.

The initial finding that OatA (formerly YjgM) could acetylate OAS was further investigated. A continuous spectrophotometric assay using DTNB was used to assess acetylation of substrates by OatA as described under “Materials and Methods” section. The specific activity of OatA for OAS was 438 nmol AMP min^-1^mg^-1^ of protein, whereas the specific activity of OatA when NAS or L-Ser was used as substrate was substantially lower, i.e., 25 and 10 nmol AMP min^-1^mg^-1^ protein, respectively (Figure 3). The same continuous spectrophotometric assay was used to determine pseudo first order kinetic parameters for the OatA enzyme with AcCoA in excess (1 mM) and varying concentrations of OAS. The data were best fit to an allosteric sigmoidal curve (*h* = 1.6) with a *K_0.5_* for OAS of 0.74 mM, the *kcat* = 1.05 s^-1^, and the catalytic efficiency (*k*_cat_/*K_0.5_*) was calculated to be 1.4 × 10^3^ M^-1^ s^-1^ (Figure 4).

### Acetylation of *O*-Acetyl-L-Serine by OatA Produces *N,O*-Acetyl-Serine (DAS)

**FIGURE 5:**
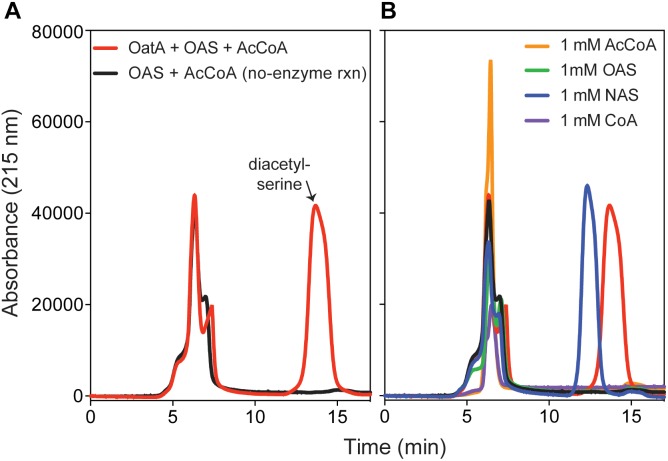
HPLC chromatograms of OatA reaction products. **(A)** Reactions containing ± OatA (80 μM), *N-*acetyl-serine (OAS, 1 mM), and AcCoA (1.2 mM) were incubated for 1 h at 37°C and OatA was removed from reaction mixture, which was then injected (100 μL) onto an Aminex HPX-87H column (Bio-Rad) as described under “Materials and Methods” section. Separation of desired product (DAS, retention time ∼14 min, red peak) was monitored using an absorbance of 215 nm. **(B)** Proper separation of DAS was compared to known standards: AcCoA (retention time between 5–7 min), CoA (retention time between 5 and 7 min), OAS (retention time 5–7 min), and NAS (retention time ∼12 min). All standards were re-suspended for a final concentration of 1 mM. Legends in **(A)** also apply to **(B)**. Experiment was repeated in three independent experiments.

**FIGURE 6:**
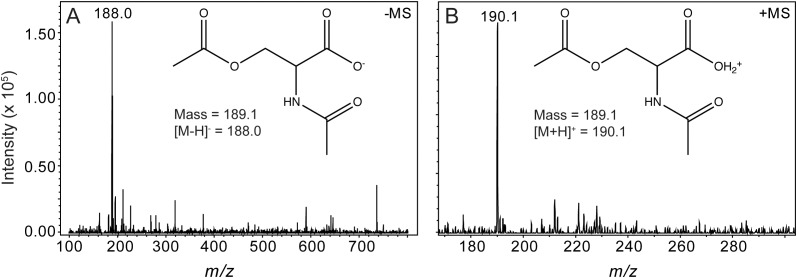
Mass spectrometry analysis of DAS, the OatA reaction product. Electrospray ionization (ESI) mass spectrometry was performed from product collected from HPLC separations (red line, peak indicated with arrow, Figure 5). Unfragmented DAS had a mass of 189.1 Da. **(A)** [MS]^-^ ionization of OatA reaction product displayed a *m/z* of 188.0 Da, which was indicative of a negatively charged DAS molecule (inset). **(B)** [MS]^+^ ionization of OatA reaction product displayed a *m/z* of 190.1 Da, which was indicative of a positively charged DAS molecule (inset). This experiment was repeated in triplicate.

We used HPLC to resolve substrates from products of the OatA catalyzed reaction (Figure 5). Under the conditions used, a peak eluted ∼14 min after injection of the sample. This peak was suspected to be DAS and was only seen when OatA was added. The retention time was different from that of NAS (12 min). The retention times of OAS and AcCoA were substantially different and overlapped at between 5–7 min after sample injection. Mass-spectrometry analysis found the compound eluting at 14 min had a *m/z* ([MS]^+^ = 190.1 Da and [MS]^-^ = 188.0 Da) corresponding to an acetyl-group on both the hydroxyl and amino group of L-Ser (Figure 6). These results, combined with the above activity assay data, indicated that OatA acetylated the amino-group of OAS producing DAS.

### OatA Is a Monomer in Solution and Binds Acetyl-CoA Before Binding OAS

**FIGURE 7:**
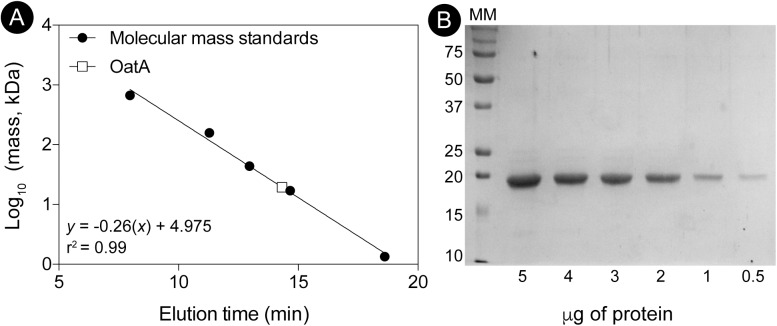
Determination of the oligomeric state of OatA using size exclusion chromatography. **(A)** The molecular mass of OatA was determined by using a Superose 12 10/300 GL size exclusion chromatography as described under “Materials and Methods” section. Molecular mass standards (black circles) were purchased from Bio-Rad with the following masses: thyroglobulin (bovine; 670,000 Da), gamma globulin (bovine; 158,000 Da), ovalbumin (chicken; 44,000 Da), myoglobin (horse; 17,000 Da), and vitamin B_12_ (1,350 Da). OatA represented as white square and molecular mass (19,500 Da) was calculated using equation displayed on graph (*r*^2^ = 0.9928). Predicted molecular mass for OatA is 18,329 Da. **(B)** Percent purity of OatA was determined by SDS-PAGE and densitometry. MM; molecular marker with relevant sizes listed (Bio-Rad Precision Plus Blue Protein Standard). Various amounts of OatA (5, 4, 3, 2, 1, and 0.5 μg) were boiled and loaded onto gel. Percent purity was calculated using Total Lab (TL100).

**FIGURE 8:**
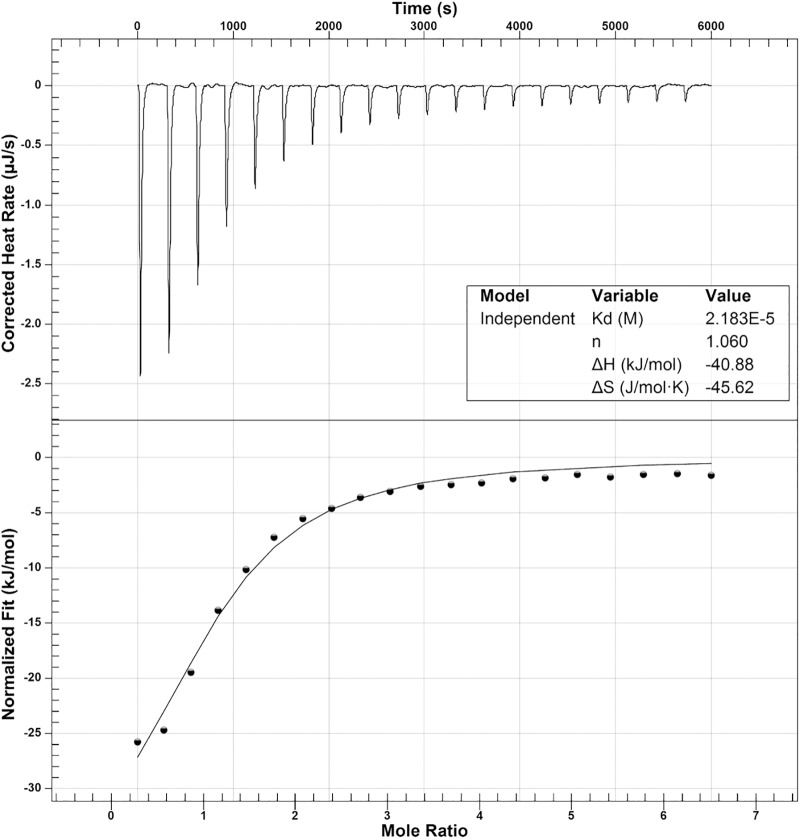
Isothermal titration calorimetry analysis of OatA binding to acetyl-CoA. ITC was used to determine binding of AcCoA to OatA. Top panel is the raw data of heat release from 20 consecutive 2.48 μL injections of AcCoA (1 mM) into a 170-μL cell containing OatA (50 μM). Bottom panel represents binding isotherms obtained by integrating the areas under the curve of each injection peak. Data were acquired using a NanoITC (TA Instruments) and data were analyzed using NanoAnalyze 1.2 software. Molar ratio of AcCoA to OatA was 1:1 (represented by *n*) and dissociation constant (*K_d_*) was found to be 2.18 × 10^-5^ M.

To determine the oligomeric state of OatA in solution, purified OatA protein was analyzed by size exclusion FPLC. Under the conditions tested, OatA eluted with a retention time consistent with that of a protein whose mass was ∼20 kDa (Figure 7A). Since the molecular mass of OatA was calculated to be 18.4 kDa, it was inferred that OatA was a monomer in solution. The purity of OatA was assessed to be 99% pure by SDS-PAGE and densitometry measurements (Figure 7B). Dissociation constants of OatA for AcCoA and OAS were determined using isothermal titration calorimetry. We found that OatA had a *K*_d_ for AcCoA of ∼22 μM with an *n*-value corresponding to one molecule of AcCoA binding per monomer (Figure 8). OatA did not bind OAS under conditions tested, suggesting that OatA may bind AcCoA before binding OAS.

### Overexpression of *oatA* in *S. enterica* Reduces the Lag Phase of Cultures Growing Under Sulfur Limiting Conditions

**FIGURE 9:**
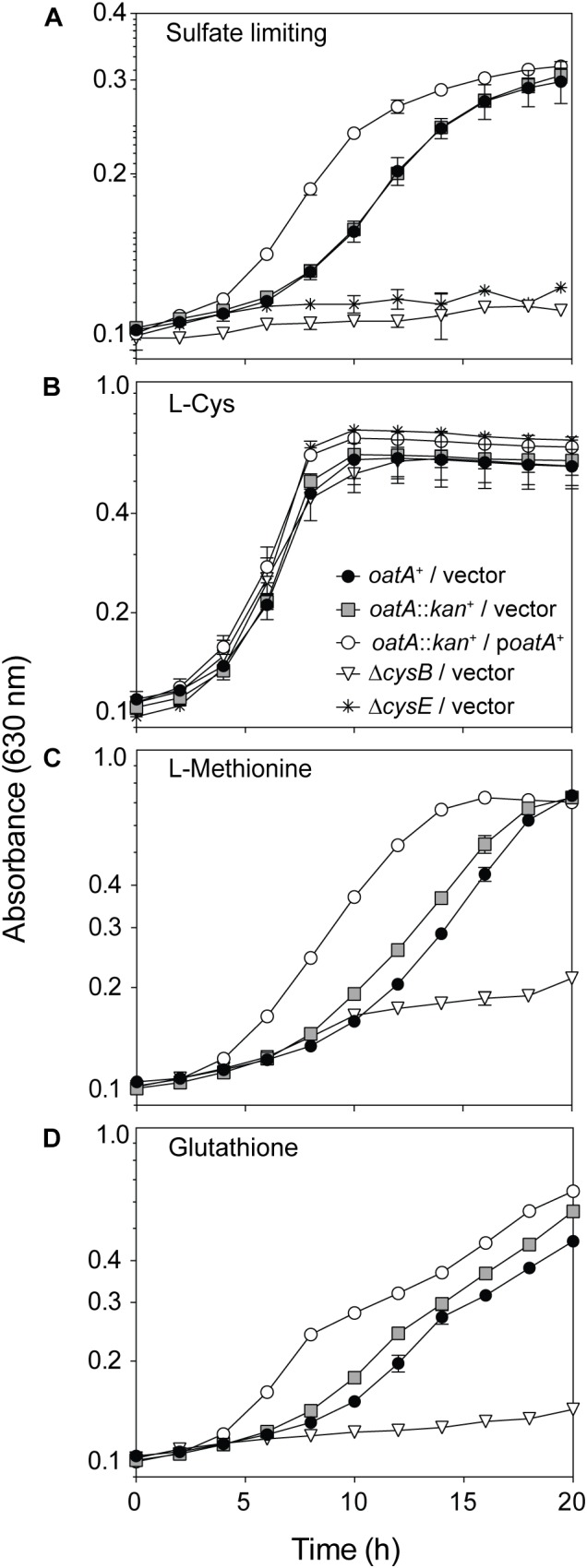
Growth analysis of cells overexpressing *oatA* under sulfur-limiting and exogenously provided cysteine conditions. The *oatA^+^* allele was cloned into a complementation vector and introduced into an *oatA*::*kan*^+^ background. The same empty vector was introduced into strains as indicated in symbol legends. Cells from overnight cultures (lysogeny broth) were washed twice and grown on minimal medium with glycerol (22 mM), chloramphenicol, and transcription of plasmids was induced by addition of arabinose (1 mM). Growth of strains was assessed under sulfate-limiting conditions (MgCl_2_ added in lieu of MgSO_4_, 1 mM) and curves were obtained using a microplate reader (BioTek Instruments). Error bars represent standard deviation and each experiment was completed in three independent experiments with strains in triplicate, with a representative graph shown. **(A)** Growth of strains under sulfur limiting conditions (MgCl_2_ added in lieu of MgSO_4_, 1 mM). **(B)** Growth after addition of exogenous L-Cys (200 μM), **(C)** L-Met (300 μM), and **(D)** oxidized glutathione (2.5 mM). Symbols in **(B)** apply to data in **(A–D)**.

To probe for OatA activity *in vivo*, a plasmid encoding an a L-(+)-arabinose-inducible promoter controlling *oatA* expression was introduced into a strain carrying a chromosomal deletion of *oatA*. Overnight cultures were washed to remove excess sulfur sources; washed cells were used to inoculate fresh minimal medium containing MgCl_2_ in lieu of MgSO_4_ (Figure 9A). These conditions were used because sulfate binds CysB and represses the *cys* regulon. We therefore used sulfate-limiting conditions to assess the potential effects of OatA-dependent OAS acetylation during growth on minimal medium, where synthesis of L-Cys was required. Any sulfate remaining in the medium was due to carry over from overnight cultures. To our knowledge, OAS is exclusively generated *in vivo* as a precursor for L-Cys biosynthesis, so we predicted OatA might be involved in this process (Kredich, 2008). When sulfate was limiting, the lag before the onset of exponential growth of *oatA*^+^ and *oat*::*kan^+^* strains was similar (∼6 h, Figure 9A, gray squares, solid circles). In contrast, ectopic expression of *oatA*^+^ reduced the lag time by half (∼3 h, Figure 9A, open circles). These differences in lag time were abolished when L-Cys was added to the medium in lieu of MgSO_4_ (Figure 9B). Overall, the cells grew to a lower final OD under sulfate limiting conditions compared to cultures with exogenous L-Cys (Figures 9A,B). It was determined that the decreased lag time associated with the overexpression of *oatA* was most likely linked to L-Cys biosynthesis, because addition of other sulfur-containing compounds (i.e., L-Met or glutathione) did not change the growth pattern of an *oatA* strain (Figures 9C,D).

### Overexpression of *oatA* Leads to Increased Transcription of *cys* Genes

*O*-Acetyl-serine is synthesized by CysE, a serine transacetylase. OAS and sulfide are the substrates of CysK or CysM, both OAS (thiol)-lyases that are involved in the last step of L-Cys biosynthesis (Figure 1). As mentioned in the *Introduction*, OAS or NAS have been proposed to bind to the CysB regulator leading to the transcriptional activation of genes needed to synthesize L-Cys. Based on this information, we analyzed the effects of OatA and DAS on L-Cys biosynthesis *in vivo*.

**FIGURE 10:**
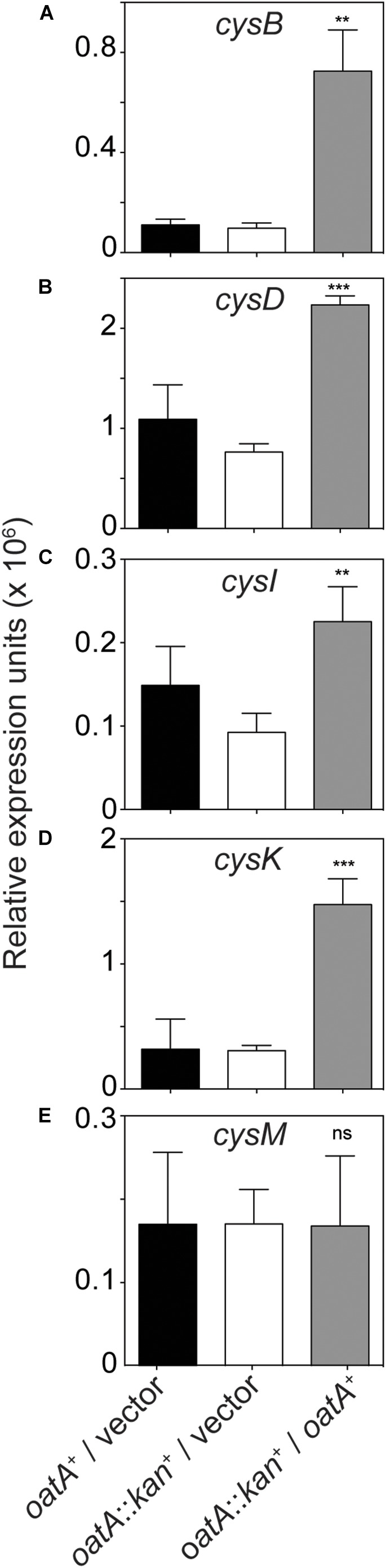
Effects of *oatA* overexpression on transcription of cysteine regulon genes. Total RNA was extracted from cells grown on glycerol (22 mM) minimal medium with MgCl_2_ (1 mM) in lieu of MgSO_4_. Cells were harvested at mid-log phase, where *oatA* overexpression caused a growth phenotype, as shown in Figure 9. Expression of *cysB* **(A)**, *cysD* **(B)**, *cysI* **(C)**, *cysK* **(D)**, and *cysM* **(E)** was assessed using RT-qPCR and transcripts were normalized to the *gyrB* gene of *S. enterica*. Changes in transcription when *oatA* was overexpressed (induction with 1 mM L-arabinose) were observed *cysB* (fivefold), *cysK* (fivefold), *cysD* (threefold), and *cysI* (threefold). Transcription was not affected with relation to *cysM*. Experiment was completed in three independent experiments, with RNA harvested in biological triplicate for each experiment and RT-qPCR completed in technical triplicate for each RNA replicate. Values shown are averages of biological triplicates with error bars representing standard deviation. *P*-values were calculated and represented by ^∗∗^ < 0.005 and ^∗∗∗^ < 0.0005.

To determine whether the shorter lag time of cultures of strains overexpressing *oatA* was due to increased levels of effector, RT-qPCR was performed under sulfur limiting conditions, for the same reasons listed above. Results from these studies showed that transcription was increased for nearly all genes known to be positively regulated by CysB upon binding of DAS (Figure 10). That is, transcription increased for *cysB* (fivefold), *cysK* (fivefold), *cysD* (threefold), and *cysI* (threefold), but *cysM* transcription was not affected (Figure 10). CysB has not yet been shown to directly regulate *cysM* and may be the reason differences were not seen.

### DAS Does Not Change CysB Binding Patterns *in vitro*

To determine effects of DAS on CysB binding *in vitro*, we purified *cysJIH* and *cysK* promoter fragments containing a 5′-6FAM fluorescent label and incubated CysB with or without DAS. The promoters of *cysK* and *cysJIH* were chosen due to changes in transcription reported in Figure 10, and because CysB binding to these promoters has been extensively characterized (Monroe et al., 1990; Hryniewicz and Kredich, 1994). OatA reactions were set up as described under “Materials and Methods” section, with a negative control reaction mixture that contained OatA but lacked AcCoA. OatA was removed from reaction mixtures and OatA products were added to EMSA reactions containing CysB and labeled probes of promoters of interest. We found that DAS altered the binding of CysB to *cysJIH*, although the effects were small and therefore determined to be non-significant (data not shown). Binding of CysB to the *cysK* promoter was not altered by DAS. Reasons for these observations are discussed below. Isothermal titration calorimetry was attempted but was not informative due to solubility issues with CysB, which has been reported (Tyrrell et al., 1997).

## Discussion

### OatA (Formerly YjgM) Is a *Bona Fide O*-Acetyl-Serine *N*-Acetyltransferase That Produces *N,O-*Diacetyl-Serine

As shown by activity and kinetic measurements (Figures 3, 4) and mass spectrometry analysis (Figure 6), OatA acetylates the free amino group of OAS yielding DAS (Figure 2). This activity is specific for OAS, because OatA failed to acetylate NAS or L-Ser *in vitro* (Figure 3). This study is the first example of enzymatic production of DAS.

### Overexpression of *oatA*^+^ Stimulates Growth of *S. enterica* on Sulfate-Limiting Medium due to Increased Expression of the *cys* Regulon

Under sulfate-limiting conditions, it has been proposed that CysB binds to NAS triggering the expression of the *cys* regulon. Several pieces of evidence reported herein challenge the idea that NAS is the sole signal controlling L-Cys biosynthesis. We proposed that under physiological conditions that trigger the expression of the *oatA* gene, the OatA *N-*acetyltransferase synthesizes *N,O-*diacetyl-serine (DAS) from OAS (Figures 3–6), and that DAS increases the expression of the *cys* regulon (Figure 10). Under sulfate-limiting conditions, one can see that cultures overexpressing *oatA^+^* enter their exponential growth phase in half the time of cultures not overexpressing *oatA*^+^ (Figure 9). The effect of DAS is specific for cysteine biosynthesis, since the addition of L-Cys to the medium mimics the effect of DAS on cell growth (Figure 9). Notably, the addition of other sulfur containing compounds such as L-Met and glutathione did not substitute for L-Cys (Figure 9). Additional support for the idea that DAS affects the synthesis of L-Cys was obtained by results of RT-qPCR analyses, which showed OatA-dependent synthesis of DAS strongly stimulated the transcription of the *cys* regulon (Figure 10).

Unfortunately, due to solubility issues with CysB^EBD^ we could not unequivocally show binding of DAS to CysB, hence raising the possibility that the positive effects of DAS on *cys* regulon expression may be indirect. At present, the question of how DAS exerts its effects remains unanswered.

### What Is the Physiological Role of OatA?

Deletion of *oatA* is not detrimental to *S. enterica* under normal laboratory growth conditions. Therefore, we would argue that the synthesis of DAS may be in response to some as-yet-unidentified environmental cue. It is possible that OatA synthesis occurs in response to some kind of stress or lifestyle change. Since there is a strong correlation between L-Cys levels and redox homeostasis, it is possible that when the latter is unbalanced additional L-Cys biosynthesis may be required, hence triggering OatA activity to synthesize DAS and increase the intracellular level of L-Cys. We note that RNA-seq analysis reported by others studying the role of CsgD in biofilm formation by *S. enterica* showed that *oatA* (formerly *yjgM*) was upregulated during planktonic growth compared to biofilm formation (MacKenzie et al., 2015). Related to these data are the findings by Sturgill et al. (2004), who reported that *cysE* (encodes serine *O-*acetyltransferase, Figure 1) mutants of *E. coli* and *Providencia stuartii* display increased rates of biofilm formation relative to the *cysE^+^* strains. Sturgill et al. (2004) proposed the existence of a CysE-dependent signal that affected biofilm formation, but the chemical nature of the compound was not identified. Based on their findings, the authors hypothesized that the hypothetical signal could be a derivative of OAS, and among the derivatives they mentioned was DAS. Additional studies in *Vibrio fischeri*, identified *cysK* transposon mutants that were deficient in forming biofilms, and the authors showed that L-Cys biosynthesis was required for biofilm formation (Singh et al., 2015). We are now in the position of testing the possibility that DAS does play a role in biofilm formation by *S. enterica.* Experiments testing this idea are ongoing. Regardless, the data reported herein provide experimental support for the biochemical activity associated with a putative GNAT *N-*acetyltransferase in *Salmonella enterica*, providing insights into the importance of acetylation in cellular physiology.

## Author Contributions

CV designed and performed all experiments, acquired and analyzed data. JE-S conceived the project, designed experiments, and analyzed data. Both authors wrote the manuscript.

## Conflict of Interest Statement

The authors declare that the research was conducted in the absence of any commercial or financial relationships that could be construed as a potential conflict of interest.
